# Prof. Dunglison

**Published:** 1847-09

**Authors:** 


*Prof. Dunglison.*—The following deserved tribute to Prof. Dunglison by one of the Editors of the St. Louis Med. and Surgical Journal, may be perused with advantage by those who would avail themselves of the secret of attaining a like position of eminence and usefulness. It is imputed to Hogarth that on some reference to his *genius*, he replied, "Genius, I know not what it is if it be not industry." That differences do exist as respects general and special mental endowments, we do not deny, but talent without application can accomplish but little in any department of effort, and unremitting industry will often compensate for the want of extraordinary natural parts. As the writer observes, Dr. D. is not to be regarded as a man of ordinary ability, yet, as he himself declares, it is by means of systematic and constant industry that he has been able to perform, and with apparent ease, such a vast amount of intellectual labor.

"We have long regarded Dr. Dunglison as a remarkable example, of one who accomplishes more in the way of science, and of professional and general literature; and has more time to devote to the cultivation of polite letters, and to the demands of society and of his family, than any one within the whole circle of our acquaintance. We speak from knowledge derived from a personal acquaintance with his habits, now some six or seven years ago—at the time Dr. Dunglison was professor (as he still is,) in one of the first Medical Colleges in the country, and filled a most important chair with distinguished ability—was visiting physicians to the Philadelphia Hospital, in which institution he delivered clinical lectures to a small class of private pupils during the summer, and to a large class from the college in the winter—was constantly issuing from the press new and standard medical works, as well as fresh editions of former publications—conducted a medical periodical—attended to his private practice—was also a prominent member of the American Philosophical Society, and of the Academy of Natural Sciences, as well as similar institutions for which Philadelphia is so celebrated, and many of whose able reports he himself prepared. In addition to all this, he kept pace with the progress of our ever advancing science, and with the current literatnre of the day—attended dinner parties, and evening parties of literary men, and always found leisure for any new or extraordinary demand on his time which might arise. All this he did, and did it well. We had the curiosity on one occasion to enquire how he effected so much, and yet never seemed hurried about anything. His reply was, "by a systematic employment of those fragments of time which are usually thrown away." We mention these facts to show what system and well directed talent will accomplish. It must not be inferred, however, that we regard Dr. Dunglison as a man of ordinary ability. Far from it. But we do believe that he owes very much of his success in life to a systematic industry, and a proper application of his talents, which all must admit are of a high order. McP."

